# From Molecules to Modules: Advanced Characterization of Membrane Systems

**DOI:** 10.1002/adma.202513056

**Published:** 2025-09-12

**Authors:** Yaguang Zhu, Austin J. Booth, Jamila G. Eatman, Min A Kim, Yining Liu, Bidushi Sarkar, Bratin Sengupta, Xiaolin Yue, Chibueze V. Amanchukwu, Seth B. Darling, Jeffrey W. Elam, Paul Fenter, Chong Liu, Kelsey B. Hatzell

**Affiliations:** ^1^ Andlinger Center for Energy and the Environment Princeton NJ 08540 USA; ^2^ Department of Chemical and Biological Engineering Princeton University Princeton NJ 08540 USA; ^3^ Chemical Sciences and Engineering Division Argonne National Laboratory Lemont IL 60439 USA; ^4^ Pritzker School of Molecular Engineering University of Chicago Chicago IL 60637 USA; ^5^ Applied Materials Division Argonne National Laboratory Lemont IL 60439 USA; ^6^ Northwestern Center for Water Research Northwestern University Evanston IL 60201 USA; ^7^ Department of Mechanical and Aerospace Engineering Princeton University Princeton NJ 08540 USA

**Keywords:** characterization, low‐dimensional materials, membranes, synchrotrons

## Abstract

Membrane technologies can enhance the efficiency and selectivity of chemical separations in energy‐water systems. Advanced characterization tools are critical for discerning separation mechanisms, revealing degradation processes, and designing novel materials and material systems for new and emerging challenges. The pursuit of next‐generation membranes for water and energy applications requires understanding phenomena at the molecular scale, mesoscale, and macroscale. This perspective highlights advanced characterization techniques for elucidating and enhancing membrane performance, while addressing fundamental trade‐offs involved in characterizing membranes under realistic conditions.

## Graphical abstract

Membrane technologies can enhance the efficiency and selectivity of chemical separations in energy‐water systems. Improving our understanding and design of separation membranes will require studying their properties at the molecular scale, mesoscale, and macroscale. This perspective highlights advanced characterization techniques for revealing membrane properties and performance across length scales and discusses the challenges involved with characterizing membranes under realistic conditions.

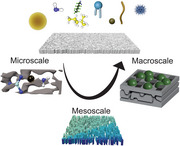

## 1 Introduction

Membrane technologies play a vital role across a broad spectrum of energy and water applications, including desalination,^[^ ^1^, ^2^ ^]^ wastewater treatment,^[^ ^3^ ^]^ battery systems,^[^ ^4^ ^]^ and membrane‐based fuel cells and electrolyzers.^[^ ^5^ ^]^ Membrane separations offer numerous advantages over existing separation methods, including energy efficiency, low chemical usage (sustainability), and scalability. Membrane techniques are also a promising approach for recovering or removing trace components from natural water sources and wastewater, as these tasks are often difficult or impractical to achieve using thermal separation methods. This difficulty arises because many water sources are complex solutions containing multiple species (e.g., various molecules and ions). Membrane selectivity is an attractive approach for targeting the chemical inactivation of viruses and bacteria,^[^ ^6^ ^]^ removing organics and oils,^[^ ^7^ ^]^ and recovering valuable resources such as geopolitically constrained lithium.^[^ ^8^ ^]^

Over the past decade, researchers have increasingly focused on advanced materials, processing methods, and synthesis strategies to enable the rational design of membranes with improved permeability, selectivity, and durability. Significant efforts have targeted a broad range of material classes, including polymeric membranes,^[^ ^9^ ^]^ inorganic membranes,^[^ ^10^, ^11^ ^]^ and composite material systems. Specific attention and efforts have focused on making membranes with i) a relatively narrow pore size distribution and high porosity,^[^ ^12^, ^13^ ^]^ ii) high mechanical stability for operation at elevated pressures, and iii) high chemical stability for longer membrane lifetimes and harsh operating conditions. Characterizing novel membrane materials and elucidating their structure–property–performance relationships are essential goals for advancing membrane science and understanding the origins of selectivity–permeability trade‐offs.

Enhancing membrane technologies for energy‐water systems also requires a deeper understanding of how local interactions within chemical microenvironments affect membrane separation performance. Separations in the energy‐water research space involve widely varied solute species that can alter separation effectiveness by interacting with membrane materials and other solutes. For this reason, another important research goal for membrane characterization is elucidating interactions between membranes and solutes in a variety of separation environments (high pressure, high temperature, complex mixtures, etc.). Fundamental understanding of how water and ion diffusion are influenced by charged moieties or varying degrees of confinement remains limited, yet these phenomena govern key transport dynamics across a range of membrane‐based technologies. Gaining insight into these mechanisms, especially under realistic rather than idealized conditions, represents a significant challenge in the field.

**Figure 1:**
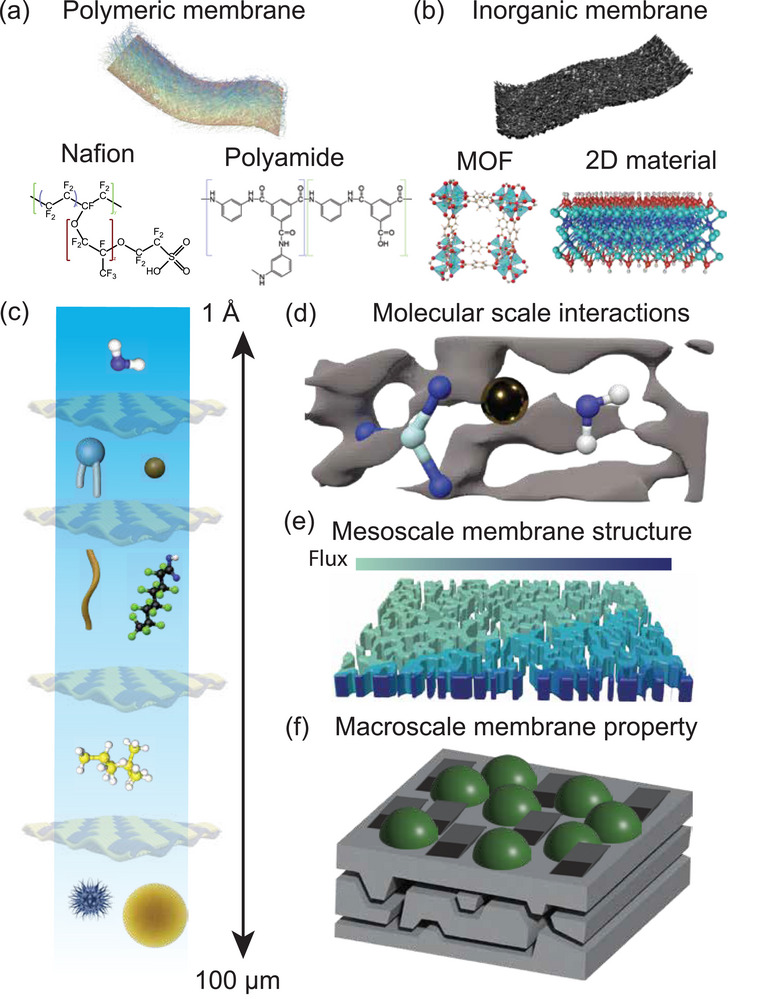
Overview of diverse chemical, dimensional, and structural features of membranes for energy‐water systems that can be revealed using advanced membrane characterization. a) Representative polymeric membrane materials: Nafion and polyamide membranes. b) Representative inorganic membrane materials, whose building units are metal‐organic frameworks (MOFs) and 2D materials. c) Importance of separation at different length scales. d–f) Novel characterization techniques can reveal critical molecular‐scale information, such as ion‐water interactions (d); mesoscale information, such as structural heterogeneity (e); and macroscale information, such as degradation and fouling (f).

To overcome limitations in selectivity, permeability, and long‐term stability, research in membrane science has focused on the development of advanced materials with tailored physicochemical and structural functionalities. Advanced membrane materials include state‐of‐the‐art polymers (e.g., Nafion and polyamide),^[^ ^14^ ^]^ polymers of intrinsic microporosity (PIMs),^[^ ^15^ ^]^ metal‐organic frameworks (MOFs), covalent organic frameworks (COFs), and 2D materials (**Figure** **1**a,b).^[^ ^1^, ^10^, ^16^, ^17^ ^]^ Polymeric membranes are engineered from an extensive library of polymeric materials, enabling precise control over pore size through diverse fabrication techniques to accommodate a wide range of separation applications.^[^ ^1^ ^]^ In contrast, novel membranes based on MOFs/COFs and 2D materials utilize the intrinsic nanopores and nanochannels in the constituent materials for separation. For example, 2D materials consist of few‐atom‐thick layers and comprise several classes of materials, including phyllosilicates (e.g., vermiculite), transition metal dichalcogenides (e.g., MoS_2_), and MXenes (e.g., Ti_3_C_2_T_*x*_).^[^ ^17^, ^18^, ^19^ ^]^

In this perspective, we explore the latest advancements in membrane characterization tools. These tools often encounter inherent trade‐offs between temporal and spatial resolution, requiring careful consideration of resolution limits and experimental design. Experimental design considerations include field‐of‐view, sample size, and separation environment. Significant knowledge gaps persist across multiple length scales, ranging from the molecular level to the bulk or macroscale. Some characterization techniques are often performed in an environment that is completely different from the one under which the membrane operates. These factors are often overlooked while interpreting such data. This perspective is structured to specifically address the tools and methods available for studying membranes at the molecular, mesoscale, and macroscale levels (Figure 1c–f).

## 2 Molecular Scale Membrane Characterization

### 2.1 Molecular Scale Phenomena in Membranes

**Figure 2:**
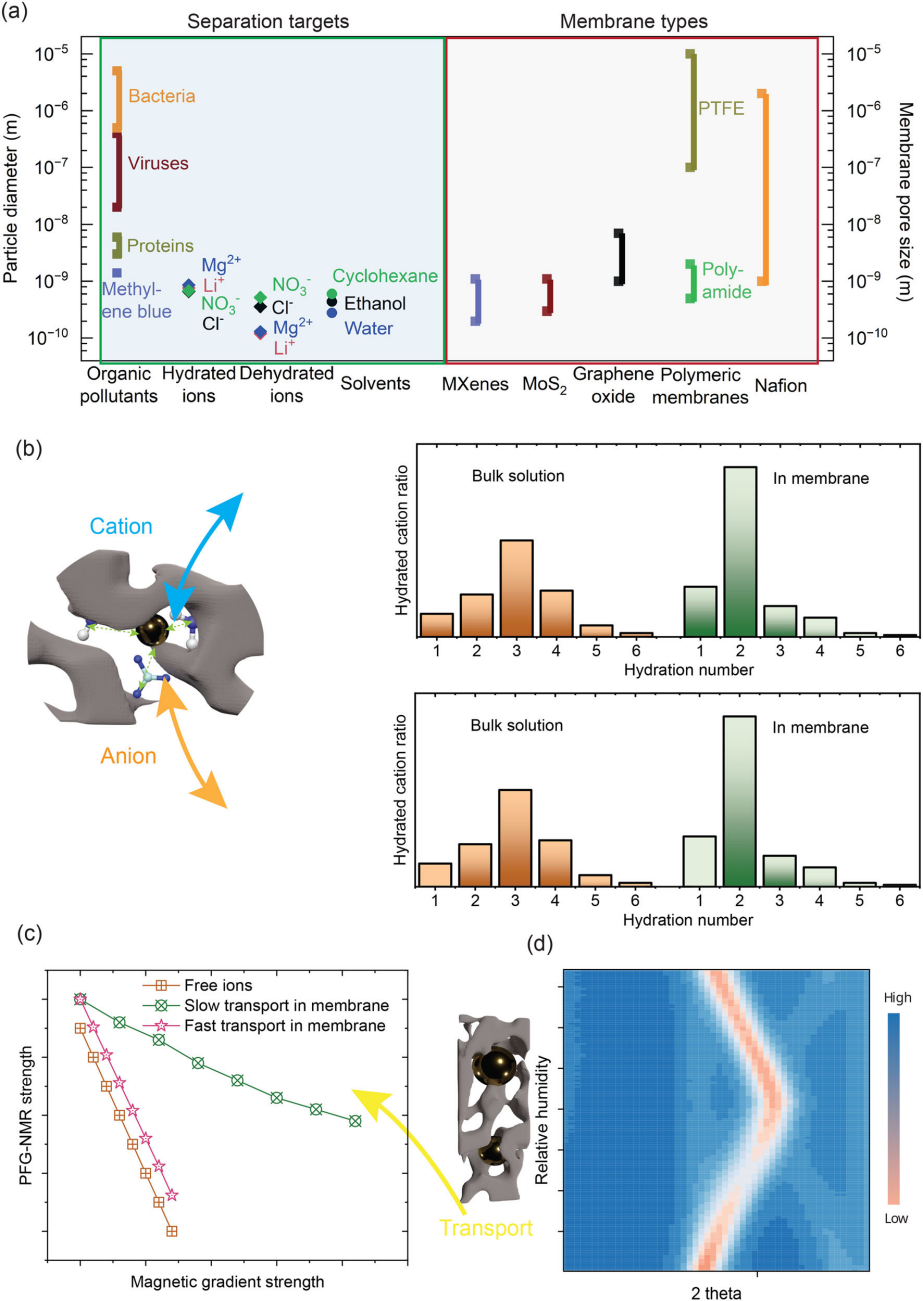
Ion‐water‐membrane interactions at the molecular scale. a) Characteristic size comparison between solutes and membrane pores. b) An example of TOF‐SIMS measurements can determine the hydrated ion structure inside the membrane. c) Example of PFG‐NMR measurements elucidate ion transport behavior inside the membrane. d) Example of water‐induced molecular 2D material membrane structural change revealed by XRD.

The size of the constituent solutes (ion, molecule, virus, etc.) can influence a separation process. Size exclusion and/or sieving separation processes are often sufficient for removal of micron‐sized particulates and viruses (Figure 1c).^[^ ^20^ ^]^ However, ions (especially those with similar charge) are more challenging to separate using conventional membrane systems, as size exclusion becomes ineffective at the angstrom scale due to the minimal size differences between ions. Properties that govern chemical microenvironments in membranes, such as surface charge and surface functional groups, can significantly impact local interactions that drive separation processes.^[^ ^11^, ^21^ ^]^ The efficiency, selectivity, and permeability of membrane separation processes depend on how ions and water molecules interact with the membrane surface (**Figure** 1c and **2**a).

Kinetic models based on transition state theory have demonstrated several key contributors to the transmembrane energy barrier.^[^ ^21^, ^22^ ^]^ However, it remains an open question whether partitioning into the pore entrance (which involves dehydration) or diffusion (including intrapore interactions) dominates transmembrane ion transport in various membrane materials. Novel membranes, including 2D‐material‐based membranes, can contain well‐defined pores or nanochannels on the scale of a few nanometers or even angstroms. Under such confinement, anisotropic solvation of ions results in complex hydration conformation and distribution, which is hard to predict using bulk hydration parameters like hydrated radius and hydration energy.^[^ ^23^ ^]^ Moreover, water's interactions with and structure near complex heterogeneous surfaces remain poorly understood. Even less is known of interfacial water at surfaces in complex aqueous fluids (e.g., concentrated ionic solutions) relevant to membrane filtration applications. For example, the ion‐water separation capability of a layer‐stacked 2D material membrane can be significantly limited by its natural tendency to swell, that is, absorb water into the nanochannel and form an enlarged interlayer spacing.^[^ ^16^ ^]^ Thus, it is important to observe the molecular structure of membranes in the wet state.

### 2.2 Novel Membrane Characterization Tools at the Molecular Scale

Numerous theoretical simulations suggest that ion dehydration is critical for transport across single‐digit and sub‐nanometer pores and that it can be affected by the size, shape, and binding energy of the solvation shell.^[^ ^24^, ^25^ ^]^ Recently, the development of a novel microfluidic chip enabled the in situ measurement of ion solvation numbers in polymeric membranes through time‐of‐flight secondary ion mass spectrometry (TOF‐SIMS).^[^ ^26^, ^27^ ^]^ TOF‐SIMS operates by bombarding a sample surface with a primary ion beam (in this case, Bi^3+^), which induces the emission of secondary ions that are then analyzed in a mass spectrometer. The results showed that cations generally could not hold more than two water molecules during sterically limited transport through nanofiltration membranes (Figure 2b).^[^ ^26^ ^]^

The fact that ionic solutions confined in hydrophobic nanospaces tend to form an incomplete dehydration structure has also been corroborated by X‐ray absorption spectroscopy (XAS).^[^ ^28^ ^]^ A recent XAS work highlights that vanadyl (VO^2+^) counter‐ions maintain a similar oxidation state, electronic state, and coordination number inside charged polymer membranes as in aqueous solutions of vanadyl sulfate, so vanadyl dehydration is unlikely in these membranes.^[^ ^29^ ^]^ These studies emphasize that different cations and anions experience greatly different hydration/dehydration processes in varying membrane environments.

Molecular‐scale transmembrane permeation includes several steps, including bulk solution diffusion, the partitioning process, rotational and translational diffusion dynamics, and bulk transport within the membrane. This complexity cannot be captured using only the solution‐diffusion, pore flow, or Nernst–Planck models.^[^ ^22^ ^]^ Several advanced techniques can provide direct measurement of ion transport within membranes. First, pulsed‐field gradient–stimulated‐echo nuclear magnetic resonance (PFG–NMR) experiments revealed a Na^+^ diffusion coefficient of 1.18 × 10^−9^ m^2^ s^−1^ in a covalent triazine framework membrane, similar to the value in pure water (Figure 2c).^[^ ^30^ ^]^ Pulsed‐field gradient NMR techniques have also been shown to be an effective strategy for discerning translational diffusional coefficients for water and sodium with ion‐exchanged polyamide membranes.^[^ ^14^ ^]^ Increasing the salt concentration in a solution has been shown to influence the concentrations of both bound and unbound protons, as well as the rotational motion of water molecules. Since water mobility can directly affect the selective permeation of different species in a membrane system, understanding these dynamics is essential for effective membrane design. Second, electrochemical impedance spectroscopy (EIS) successfully differentiated between ion transport in electrical double layers at the membrane surface versus transport in the diffusion boundary layers arising from the interface between the ion‐exchange membrane and the electrolyte solution.^[^ ^31^ ^]^ Third, quasielastic neutron scattering (QENS) evaluated the effect of water (degree of humidity) on the local water and proton dynamics in Nafion membranes.^[^ ^32^ ^]^

For 2D‐material‐based membranes, the nanochannel size and the size of in‐plane pores directly correlate with resistance to ion and water transport. Importantly, the nanochannel size can be actively modulated by ions. For example, intercalation of Li^+^, K^+^, Na^+^, Rb^+^, Mg^2+^, and Ca^2+^ cations can result in different nanochannel size in MXenes and other 2D materials.^[^ ^33^ ^]^ Additionally, in situ X‐ray diffraction measurements revealed that the nanochannel size in MXene and graphene oxide membranes can change in response to changes in humidity (Figure 2d).^[^ ^16^, ^34^ ^]^ Ambient pressure X‐ray photoelectron spectroscopy can track local membrane hydration and dehydration processes by introducing a high humidity environment and modulating temperature.^[^ ^35^ ^]^ Hydrophobic (K^+^) cations decrease water mobility within the confined interlayer and accelerate water removal at nonconfined surfaces.^[^ ^35^ ^]^ Hydrophilic cations (Li^+^) increase water mobility within the confined interlayer and decrease water‐removal rates at nonconfined surfaces.^[^ ^35^ ^]^

Understanding chemical microenvironments in a wide range of membrane‐relevant material systems remains a challenge due to the limitations of existing characterization techniques. Many approaches require ultra‐high vacuum conditions, have limited fields‐of‐view that hinder statistical analysis, or use probes such as electron beams that can damage or destabilize sensitive materials. Recent studies have reported anomalous permeation behavior for Pb^2+^ and Cu^2+^ ions through MoS_2_‐based membranes.^[^ ^36^ ^]^ Bulk uptake experiments showed that these membranes exhibited a greater affinity for these ions compared to other divalent cations such as cadmium and magnesium. The fast entrance kinetics and narrow interlayer spacing of Pb^2+^ can expel other ions by raising their entrance barriers. High‐angle annular dark‐field scanning transmission electron microscopy (HAADF‐STEM) enabled direct visualization of these ion‐material interactions, revealing that ion‐MoS_2_ interactions do not change the material framework of the membrane but modify local structures.^[^ ^36^ ^]^ In addition, infrared spectroscopy has provided insight into how ions coordinate with specific surface moieties.

Visualizing membranes in a hydrated state remains a challenge for a range of X‐ray and electron imaging tools. Atomic force microscopy and quartz crystal microbalance techniques provide some spatial resolution and insight into material properties, but they are limited to surface‐level characterization and cannot capture 3D structures. Recently, cryo‐TEM tomography has emerged as a powerful tool capable of visualizing polyamide reverse osmosis membranes in their hydrated state.^[^ ^37^, ^38^ ^]^ This 3D imaging approach enables quantitative analysis of structural changes that occur in the membrane upon hydration. In practice, a combination of complementary techniques is often necessary to fully resolve both structural and chemical aspects in membrane systems. Linking observations across multiple experimental techniques often benefits from complementary modeling to enhance the interpretation of complex datasets.

### 2.3 Challenges and Opportunities for Molecular Scale Membrane Characterization

Measuring and tracking molecular‐scale interactions between water, ions, and membrane surfaces is essential for understanding membrane behavior, but remains challenging due to the inherent heterogeneity of these interactions at the nanoscale. First, it is necessary to distinguish chemical information involving water and ions at different locations in the membrane. For instance, hydration layers at the membrane surface exhibit distinct properties compared to bulk water, but most experimental techniques average out these effects. Advanced spectroscopy methods, such as sum‐frequency generation (SFG), provide surface‐specific insights on water structure.^[^ ^39^ ^]^ A careful interpretation of overlapping vibrational modes can resolve distinct structures of water and ions at the water‐membrane interface. As a second‐order optical process, the sign of the output SFG response is dependent on the molecular orientation, making any signals from bulk solution (isotropic environments) vanish. Thus, sum frequency generation vibrational spectroscopy is an emerging technique in the membrane field to defect the orientation of water molecules at the interface. Second, many membranes exhibit nanoscale heterogeneities in charge distribution, porosity, and functional group density.^[^ ^17^, ^40^ ^]^ These variations influence local ion transport and hydration behavior. Liquid‐cell transmission electron microscopy provides new opportunities to study the molecular structure of membranes immersed in water and accompanied by multiple ion species.^[^ ^41^ ^]^ For example, such techniques can directly visualize nanochannel swelling in 2D‐material‐based membranes and be correlated with ion or water intercalation events.^[^ ^16^, ^42^ ^]^ In addition, combining spectroscopy techniques with high‐resolution structural characterization can help capture dynamic heterogeneities in membrane systems which contribute to transport mechanisms.^[^ ^43^, ^44^ ^]^

In real‐world applications, membranes interact with complex electrolyte solutions rather than a single ion. In this complex matrix, multiple modeling works have suggested the formation of ion pairs and highlighted their great importance in modulating ion transport behavior.^[^ ^45^, ^46^ ^]^ Atomic pair distribution function (PDF) and extended X‐ray absorption fine structure (EXAFS) can provide information on local atomic arrangements, interatomic distances, and coordination environments.^[^ ^47^ ^]^ Computational approaches such as molecular dynamics and artificial intelligence can also provide broad insight into ion transport behavior and membrane properties at the molecular scale.^[^ ^48^, ^49^, ^50^ ^]^ Operando X‐ray diffraction and absorption characterization can bridge the gap between experimental measurements and computational modeling; these techniques will be critical in designing next‐generation membranes with precise molecular control over ion transport and rejection.

## 3 Mesoscale Membrane Characterization

### 3.1 Mesoscale Phenomena in Membranes

Accurate membrane structural characterization enables rational material design, bridging the gap between molecular‐scale interactions and macroscopic performance. Water flux and solute rejection are primarily controlled by the porosity, pore size distribution, and pore tortuosity of the membranes. Few membranes follow the Hagen–Poiseuille equation for simple fluid flow through a cylindrical pore, due to complicated pore geometries.^[^ ^51^ ^]^ For example, in polyamide membranes, transmission electron microscopy reveals pores 20 to 100 nm in size with interconnecting hollow passages surrounded by protuberances and ridges.^[^ ^37^, ^38^, ^52^, ^53^ ^]^ Therefore, dedicated characterization of both the surface and the interior of the membrane is necessary to elucidate complex membrane structure.^[^ ^17^, ^38^ ^]^

Indirect measurements of pore size distribution, such as bubble point pressure analysis, membrane impedance porosimetry, mercury porosimetry, and physisorption analysis, can only determine the largest pore entrance and cannot detect isolated pores.^[^ ^54^ ^]^ In addition, information from 2D imaging can be insufficient, while data in 3D provides more statistical information about the porous structure. For example, membranes obtained from vacuum‐assisted assembly of 2D materials will have nonuniformly distributed free volume defects, leading to inefficient permselectivity.^[^ ^17^ ^]^ Thus, instead of 2D cross‐sectional images, 3D imaging of the membrane can provide a more comprehensive investigation of membrane features.

### 3.2 Novel Membrane Characterization Tools at the Mesoscale

**Figure 3:**
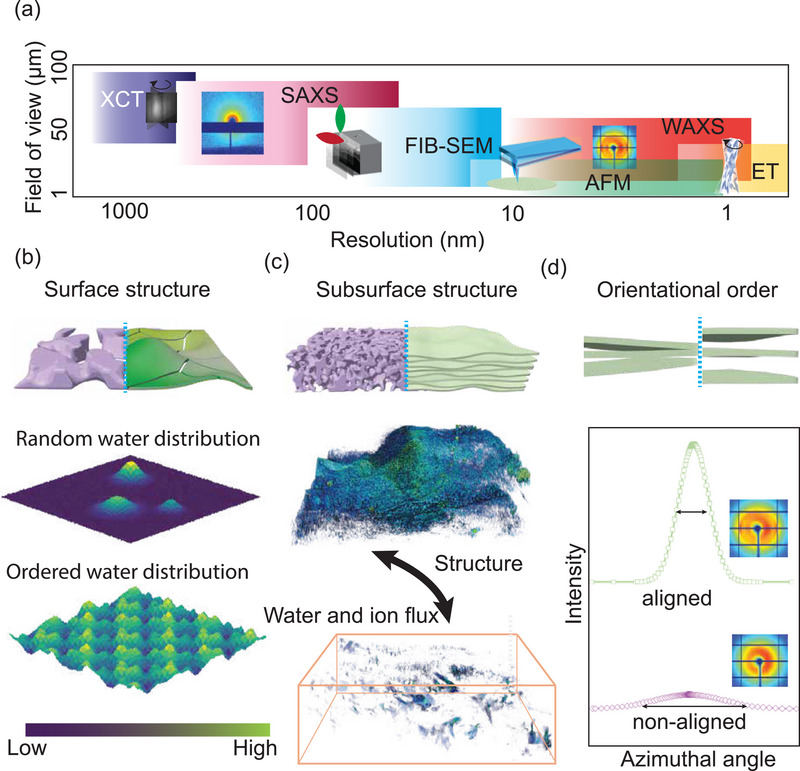
Advanced characterization of membrane structure at the mesoscale. a) Field of view and resolution of novel characterization tools. b) AFM‐IR correlates water with the surface structure of the membrane. c) Tomography tools correlate membrane structure with ion and water flux. d) X‐ray scattering tools reveal the structural orientation of membranes.

Advanced characterization techniques, including atomic force microscopy (AFM), electron tomography (ET), focused ion beam scanning electron microscopy (FIB‐SEM), X‐ray computed tomography (XCT), small‐angle X‐ray scattering (SAXS) and neutron scattering (SANS), and wide‐angle X‐ray scattering (WAXS), can probe the surface and bulk structure of membranes under different fields of view and resolutions (**Figure** **3**a). In the transmembrane transport process, ions and water first interact with the membrane surface. AFM is particularly well suited for revealing surface information, including surface roughness, surface pore size, and pore size distribution. Recent efforts have expanded AFM's capabilities by coupling AFM with infrared spectroscopy (AFM‐IR). For instance, AFM‐IR was able to probe the aggregation and distribution states of water on 2D‐material membrane surfaces, revealing differences between graphene oxide and MXene sheets^[^ ^55^ ^]^ (Figure 3b).

After water/ions partition into the membrane, the membrane structure and local chemistry actively modulate ion and water transport within the membrane. Recently, with the development of high‐angle annular dark‐field scanning TEM imaging, ET has been applied to overcome imaging artifacts in low‐electron‐density polymeric membranes and quantify their nanoscale structural and morphological characteristics.^[^ ^56^ ^]^ 3D imaging via ET can clearly show internal microstructural heterogeneity and can accurately estimate the surface area and void fraction in polyamide membranes.^[^ ^37^, ^53^ ^]^ Furthermore, scanning TEM combined with electron energy loss spectroscopy (EELS) can reveal variation in the distribution of functional groups in polyamide membranes, which is important for understanding the transport of complexed ions and water.^[^ ^57^ ^]^

3D visualization of membranes can provide not only structural information but also key topological properties. 3D visualization techniques are thus helpful for understanding transport of ions and water within the membrane (Figure 3c). For example, XCT on polymer electrolyte membranes revealed a tortuosity decrease from 5.63 to 2.82 when the pore size in the porous transport layer increased from 16 to 90 μm.^[^ ^58^ ^]^ Importantly, XCT can also directly visualize membrane swelling and shrinkage.^[^ ^59^ ^]^ Another technique, nanotomography, boasts enhanced resolution (≈30 nm) for visualizing nanoscale features in the membrane. Nanotomography can quantify properties including porosity, 3D morphology, pore interconnectivity, and tortuosity in various membrane materials.^[^ ^60^ ^]^ Alternatively, FIB‐SEM can resolve pores and defects with sizes as small as 20 nm (such as in 2D‐material membranes including graphene oxide and MXene), although it is a destructive imaging method.^[^ ^17^, ^19^, ^61^ ^]^

SAXS and WAXS can elucidate the orientation order of membranes with highly anisotropic structures (Figure 3d). For instance, 2D‐WAXS azimuthal angle measurements revealed that changing the fabrication method for MXene membranes from vacuum filtration to blade coating can significantly enhance flake alignment, which can affect Li transport pathways.^[^ ^62^ ^]^ Similarly, SAXS showed that uniaxial deformation induces alignment of ionic domains along the stretching direction in perfluorosulfonate ionomer membranes.^[^ ^63^ ^]^

Emerging tools for mesoscale characterization also include operando techniques, which can reveal structural evolution in membranes during transport and can accurately monitor pore size at the mesoscale. As an example, operando grazing incidence X‐ray scattering can provide time‐resolved data on d‐spacing; this technique has been applied to membranes (such as 2D‐material‐based membranes) to probe swelling during transport.^[^ ^64^, ^65^ ^]^ Operando SANS can also characterize pore size (and changes in pore size) during the operation of reverse osmosis membranes.^[^ ^66^ ^]^ Operando SAXS can study nano‐ and microstructural evolution (i.e., aging) in membranes.^[^ ^67^ ^]^ Additionally, the emerging technique of optical coherence tomography allows for noninvasive 3D reconstruction of membrane structures.^[^ ^54^, ^68^ ^]^ Utilizing light in the near‐infrared spectral range, optical coherence tomography can probe a depth of several hundred microns in the membrane to provide images of the membrane structure and the distribution of scales.^[^ ^69^ ^]^ An interferometric set‐up will collect the backscattered light and reconstruct the depth profile of the membrane sample at the selected location.^[^ ^70^ ^]^

### 3.3 Challenges and Opportunities for Mesoscale Membrane Characterization

Understanding pore size distribution is essential for assessing membrane performance, especially when separation depends solely on species size. However, membrane characterization is often performed under conditions that are very different from actual separation conditions. For example, TEM images of MOF/COF membranes are acquired under high vacuum (≈10^−5^ Pa), while filtration is conducted under pressure in water or solvents. Utilizing a liquid cell for transmission electron microscopy, electron tomography can also measure the membrane structure in solution, but it is challenging to conduct experiments involving pressure. Reports of MOF/COF flexibility under such conditions cast doubt on whether TEM‐observed pores accurately represent those active in separation.^[^ ^71^, ^72^ ^]^ Moreover, electron beam irradiation during TEM can alter nanomaterials, complicating imaging of in‐plane pores in 2D materials like graphene or graphene oxide, where beams may etch and enlarge defects.^[^ ^73^ ^]^ These discrepancies challenge the reliability of TEM for accurately predicting operational separation performance, highlighting the need for characterization methods that mimic real‐world conditions in practice.

N_2_ adsorption at 77 K is often used to determine a membrane's pore size distribution by capturing a complex network of pores—including dead‐ended pores—through which a molecule travels from the feed to the permeate side, sometimes encountering a single choke‐point that acts as a bottleneck to prevent the passage of a specific species.^[^ ^74^, ^75^ ^]^ However, since only a minuscule portion of these probed pores may be the actual bottlenecks responsible for selectivity, drawing deterministic evidence about the separation‐relevant pores—especially in amorphous materials with non‐ordered pores—may be erroneous. Another typical challenge faced in determining pore size distribution via adsorption isotherms is the choice of probe molecule. N_2_ isotherms are fairly accurate for materials with micropores (excluding ultra‐micropores) and mesopores (i.e., materials for loose reverse osmosis and nanofiltration membranes), but they are unreliable for ultrafiltration and microfiltration membranes due to the material's low surface area. In summary, sufficient care must be taken while analyzing and concluding from pore characterization. It is advisable that pore characteristics measured from multiple characterizations should be compared with method permeation experiments with neutral probe molecules.^[^ ^13^ ^]^

AFM is another technique that often provides a non‐destructive method for understanding membrane pore size. However, AFM is seldom conducted on membranes in the solvated state, which gives rise to discrepancies where the pore size determined via AFM conflicts with observed separation performance.^[^ ^76^ ^]^ Recently, data analysis methods have been developed for AFM images of ultrafiltration membranes that yield comparable pore sizes to more conventional FE‐SEM measurements.^[^ ^77^ ^]^

## 4 Macroscale and Multiscale Membrane Characterization

### 4.1 Macroscale Phenomena in Membranes

**Figure 4:**
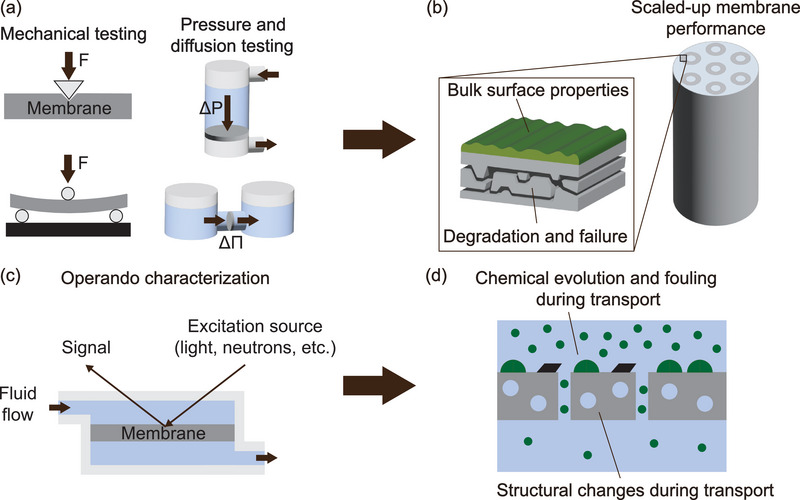
Macroscale and operando testing can reveal membrane structure‐property‐performance relationships across length scales. a) Mechanical and permeation testing can measure bulk membrane properties and performance. b) Applications of macroscale characterization include studying membrane degradation, failure, and bulk performance, both for individual membranes and for large‐scale membrane modules. c) Operando characterization can couple structural or chemical information with bulk permeation testing during membrane operation. d) Applications of operando characterization include measuring structural changes, chemical changes, and fouling during transport.

The mechanical properties of membranes play a critical role in determining their stability, durability, and practical utility. Attributes such as strength and elasticity influence both the operational lifespan of the membrane and its suitability for various applications. These properties generally pertain to the bulk material and are commonly assessed using macroscale measurements (**Figure** **4**a). Traditional macroscale mechanical characterization methods, such as tensile and flexural testing, are effective for quantifying stress–strain behavior and elastic properties in relatively brittle membrane materials.^[^ ^78^, ^79^, ^80^ ^]^ For highly elastic membrane materials, such as polymers, dynamic mechanical analysis (DMA), which evaluates viscoelastic behavior under oscillatory stress, is a more suitable characterization technique.^[^ ^78^, ^81^ ^]^ Additionally, nanoindentation can enable the measurement of elastic modulus, hardness, and fracture toughness even for small samples.^[^ ^78^, ^82^ ^]^ However, many traditional mechanical testing methods are destructive and require measurement ex situ, posing a challenge for accurate measurement of membranes' properties during operation.

Another key area of macroscale characterization involves directly measuring solute or liquid transport across the entire membrane. Macroscale permeation testing can quantify bulk transport phenomena and determine large‐scale membrane performance metrics using pressure‐driven or diffusion‐driven testing. The most common form of diffusion‐driven testing is a lab‐scale permeation cell (i.e., H‐cell), which secures a membrane between a feed chamber and a permeate chamber. The solute concentration in the permeate chamber can then be monitored over time via ion chromatography, conductivity measurements, inductively coupled plasma mass spectrometry (ICP‐MS), etc.^[^ ^13^, ^17^ ^]^ This setup can yield solute flux, permeability (i.e., flux normalized by membrane thickness), and selectivity under diffusion‐driven conditions.^[^ ^18^ ^]^ Similarly, the simplest form of pressure‐driven testing is dead‐end filtration, which uses pressure to pass the feed solution through the membrane (the solution flows perpendicular to the membrane surface) and generates a permeate solution that can be collected and analyzed. This method can measure both liquid and solute flux, as well as permeability and selectivity.^[^ ^83^ ^]^ Moreover, rejection experiments with small neutral solute molecules of varying sizes can often be translated to the membrane's pore size distribution via appropriate models.^[^ ^84^ ^]^

Macroscale characterization, including both mechanical testing and permeation testing, is a useful approach to understanding membrane degradation and its effects on performance. For instance, mechanical testing can quantify how membranes' strength and elasticity evolve over time and under different experimental conditions.^[^ ^78^, ^79^, ^80^, ^85^ ^]^ Macroscale testing combined with structural analysis or imaging techniques (either ex situ or operando) can further elucidate structure‐performance relationships in membranes. For instance, pressure testing in combination with microscopy can reveal how membrane compaction impacts performance.^[^ ^86^ ^]^ Permeation testing can also be correlated with mechanical testing results to evaluate performance‐durability trade‐offs in membranes.^[^ ^87^ ^]^ Degradation of membrane materials in water or organic solvents can also be investigated by coupling macroscale methods, including optical imaging and permeation testing, with microscopic methods such as X‐ray diffraction and zeta potential measurement.^[^ ^88^, ^89^ ^]^

### 4.2 Novel Membrane Characterization Tools at the Macroscale

Some specialized techniques have recently been developed for membrane mechanical testing. Burst testing provides a method for determining the practical mechanical strength of large‐scale membranes, such as flat sheet and hollow fiber membranes.^[^ ^78^ ^]^ The emerging technique of micromechanical spectroscopy allows accurate measurement of mechanical properties in very rough or low‐dimensional membrane samples.^[^ ^90^ ^]^ Another novel technique, hydrodynamic bulge testing, can measure elastic properties in soft materials while eliminating the error associated with directly measuring deformation.^[^ ^91^ ^]^

Macroscale characterization techniques are important for evaluating membranes' scalability (i.e., performance and challenges under industrial‐scale conditions). In industrial settings, membranes are often operated in cross‐flow, co‐flow, and counter‐flow geometries, which are more efficient than simple diffusion or dead‐end cells and experience different transport phenomena.^[^ ^92^, ^93^, ^94^, ^95^ ^]^ Lab‐ or pilot‐scale setups for characterizing membrane performance under these geometries are of particular interest for bridging the gap between research‐scale and industrial membrane performance testing. In a recent example, graphene‐based membranes (part of the rapidly growing class of 2D‐material‐based membranes) were scaled up using spray‐coating, then tested under pressure‐driven cross flow to quantify their shear resistance and chemical stability.^[^ ^96^ ^]^ Lab‐scale cross‐flow, co‐flow, and counter‐flow membrane setups have also been applied to study properties such as salt rejection, selectivity, and resistance to shear.^[^ ^97^, ^98^ ^]^

Macroscale mechanical and performance testing can also give insights into membrane scalability under even more complex geometries and larger membrane areas, including entire membrane modules (Figure 4b). For example, large‐area polymeric flat sheet membranes' longevity and stability have been studied via mechanical testing methods combined with permeation testing.^[^ ^78^, ^99^ ^]^ Macroscale testing methods have also helped reveal transport phenomena, concentration polarization, and degradation behavior in hollow fiber membranes, one of the most common membrane module geometries in industry.^[^ ^78^, ^81^, ^100^, ^101^, ^102^ ^]^

Macroscale membrane characterization is also closely related to the growing field of operando and online membrane characterization. Operando membrane characterization broadly refers to techniques that couple structural or chemical analysis with macroscale performance measurements while a membrane is being operated (Figure 4c). Operando methods are thus uniquely suited to probing membranes' properties and structure‐property‐performance relationships under realistic operating conditions. For instance, operando characterization can provide information about membrane degradation and longevity by tracking molecular‐scale structural or chemical evolution alongside changes in bulk membrane performance (Figure 4d). Operando characterization can also probe molecular transport pathways and transition states by analyzing solute chemistry as transport is occurring.

Many light‐based scattering and imaging techniques have been adapted for operando membrane characterization, particularly X‐ray‐based techniques. Several of these techniques are suitable for characterizing membrane materials' chemistry and structure during operation, such as operando X‐ray absorption spectroscopy, X‐ray photoelectron spectroscopy, X‐ray fluorescence, X‐ray diffraction, and wide‐/small‐angle X‐ray scattering.^[^ ^64^, ^65^, ^67^, ^103^, ^104^, ^105^, ^106^, ^107^, ^108^, ^109^ ^]^ In addition to X‐ray techniques, operando infrared spectroscopy, Raman spectroscopy, and neutron imaging can probe the molecular structure and chemistry of membrane materials.^[^ ^65^, ^104^, ^108^, ^110^, ^111^, ^112^ ^]^ Operando microscopy and tomography can also provide non‐destructive 3D data on membranes and foulants.^[^ ^54^, ^113^, ^114^ ^]^

Operando techniques such as those mentioned above are particularly suitable for online membrane evaluation (e.g., operando monitoring in conjunction with automated manufacturing or industrial processes). For instance, inline operando methods such as imaging, microscopy, or infrared spectroscopy could be integrated with a manufacturing line to monitor defect formation and perform quality control in real time. Additionally, operando structural and chemical analysis could be incorporated into industrial membrane modules or automated membrane testing setups, allowing for online monitoring of membrane degradation or changes in performance. These techniques will be especially important for process optimization as the membrane industry moves toward Industry 5.0.

### 4.3 Challenges and Opportunities for Macroscale Membrane Characterization

Macroscale characterization of membranes is a crucial step in understanding their performance under conditions similar to real‐world applications. However, evaluating transport properties such as permeability, selectivity, and mechanical stability at this scale presents both challenges and opportunities.

One major challenge is concentration polarization and fouling, which are well‐studied issues in size‐exclusive membrane transport. These phenomena can significantly alter rejection and flux profiles over time due to the accumulation or adhesion of solutes near or on the membrane surface. While concentration polarization effects can be mitigated by adjusting operating conditions like feed velocity and pressure, some types of fouling are irreversible due to solute adhesion within pores and on surfaces. In such cases, operational adjustments alone cannot restore flux, highlighting the need for standard procedures to assess interface dynamics and develop robust antifouling materials and operational conditions for precise separation.

As the membrane area increases, inhomogeneity in pore sizes and tortuosity can lead to unpredictable solute rejection behavior. Ensuring sample uniformity in bulk and conducting mesoscale surface characterization are critical pre‐assessment steps to predict separation performance. Characterizing membranes at the module level adds complexity due to intricate engineering flow designs. Large‐scale processes require maintenance to extend membrane lifespan and must contend with complex flow patterns and severe fouling due to module geometry.

Another key challenge in macroscale characterization is bridging the gap between laboratory‐scale methods and real‐world industrial applications. Diffusion‐based methods have been widely applied for lab‐scale characterization of membrane transport properties due to the simple experimental setup. However, the transport process is driven solely by concentration gradient and has slow kinetics, limiting its practical relevance. Electrodialysis can accelerate the transport process using an external electric field, which, while effective, is often cost‐prohibitive due to its complex setup, energy demands, and the need for specialized ion‐exchange membranes.^[^ ^115^ ^]^ Furthermore, as electro‐osmotic and diffusive transport depend on different mechanisms and occur at different rates in many materials,^[^ ^116^ ^]^ electrodialysis testing should not be used to draw generalized conclusions about transport through novel membrane materials. Instead, pressure‐driven tests are commonly used for membranes in industrial application scenarios. Lab‐scale pressure‐driven tests can be operated using cross‐flow or dead‐end filtration setups, which are common for robust polymeric membranes that can withstand high pressures, but these conditions pose significant challenges for membranes with weaker mechanical strength, such as emerging 2D‐material‐based membranes.

Simplified characterization conditions are another pitfall of laboratory‐scale methods. Many studies report membrane transport properties based on tests with a limited number of solutes, overlooking the complexities of industrial processes that often involve mixtures of known and unknown species.^[^ ^1^, ^3^ ^]^ To enhance relevance, transport properties should be characterized under conditions that simulate practical scenarios. Additionally, the impact of coexisting species on membrane structure and performance remains underexplored, despite its critical importance for optimizing membrane functionality.

Despite these challenges, macroscale membrane characterization offers significant opportunities. One promising area is bridging the gap between lab‐scale and industry‐scale applications through automation, scalability, and high‐throughput techniques. These approaches enable researchers to efficiently evaluate membrane performance during the lab‐scale development process and enhance the reliability of scale‐up predictions, facilitating industrial adaptation. Furthermore, automated workflows allow for the integration of advanced computational models and machine learning to predict membrane characteristics, such as performance under hazardous conditions, optimal design parameters, and potential failure modes. This paves the way for developing custom membranes tailored to specific applications.

## 5 Outlook

### 5.1 Applications of Coherent X‐Ray Characterization for Studying Membranes

A revolution in the study of polymeric and 2D ion separation membranes is now being facilitated by the recent development of ultra‐bright coherent X‐ray beams produced at 4th generation X‐ray sources,^[^ ^117^ ^]^ especially for high energy X‐ray beams (i.e., E >10 keV) that can readily penetrate through materials in complex environments and sample cells.^[^ ^118^ ^]^ These coherent X‐ray beams enable the application of emerging X‐ray techniques, such as X‐ray photon correlation spectroscopy (XPCS) and coherent X‐ray diffraction imaging (CXDI) to the operando study of real‐world materials, including the visualization of nanoscale membrane architectures and ion transport. CXDI addresses an ongoing challenge to obtain high‐resolution images of membrane structure by mapping nanoscale pores and lamellar channels (e.g., in MXene, MoS_2_ or graphene membranes) in their fully hydrated state. While electron microscopy has the resolution to observe such structures, it remains extremely challenging to make observations under operating conditions, because of the limited penetration of electron beams and because the spatial resolution is compromised under operando conditions (e.g., >1 µm in a liquid).^[^ ^119^ ^]^ In contrast, traditional (incoherent) X‐ray imaging approaches such as computed tomography (CT) can provide operando images of membrane structure, but its resolution (typically a few microns) is insufficient to resolve the relevant pore structure.^[^ ^120^ ^]^ CXDI bridges this gap by extending the resolution of CT approaches through “lens‐less” imaging. This is enabled by the high X‐ray beam coherence that is manifested by the presence of “speckles” in the angular distribution of scattered X‐rays which encode the actual sample structure.^[^ ^121^, ^122^ ^]^ 3D images can be obtained with resolutions as low as tens of nanometers, with fields of view of tenss of microns, through measurements performed over multiple sample orientations (e.g., coherent tomography)^[^ ^123^ ^]^ or overlapping beam positions (ptychography)^[^ ^124^ ^]^ and coupled with the associated mathematical algorithms that invert these data. XPCS measures equilibrium density fluctuations in materials.^[^ ^125^, ^126^ ^]^ When a coherent X‐ray beam interacts with the membrane, a speckle pattern will be produced. To obtain the scattering‐vector‐dependent dynamics in the membrane sample, we can calculate the average correlation of the intensity within individual speckles over time. This average correlation is directly related to surface and membrane fluctuation.^[^ ^125^, ^126^ ^]^ This approach is analogous to dynamic light scattering but with sensitivity to nm‐scale behavior due to the short x‐ray wavelength. This approach will yield unprecedented insight into ion motions, ion pathways, transport heterogeneity and the dynamic evolution of the membrane matrix. For example, multi‐modal diffusion processes or spatially varying flow rates can be discerned within a single membrane. XPCS is uniquely suited to probe such complexity, since it penetrates opaque, aqueous environments and measures fluctuations rather than just average structure. XPCS has already demonstrated the ability to probe polymer chain motion at the nanometer scale,^[^ ^127^ ^]^ flow velocities,^[^ ^128^ ^]^ and deviations from Fickian diffusion.^[^ ^129^ ^]^ It can also distinguish stationary dynamics (e.g., diffusion) from non‐equilibrium dynamics through the use of two‐time correlation functions 14. Recent studies also have demonstrated the ability to probe fast dynamics at ns time scales.^[^ ^126^ ^]^ The use of resonant contrast^[^ ^130^, ^131^ ^]^ can extend XPCS to isolate the dynamics of specific components of interest (e.g., ion, solute, or membrane matrix) by using photon energies near the absorption edge of a given element. This capability, however, will likely be restricted to elements with absorption edges in the hard X‐ray range so that the photons can penetrate through membranes (for example, the x‐ray attenuation length in water is ≈0.7 mm near the K‐absorption edge for Fe at 7.11 keV). A high‐energy X‐ray beam can penetrate the membrane and measure its structural and chemical information when the membrane is fully immersed in the solution. By capturing nanoscale structure and dynamics simultaneously, these coherent X‐ray methods offer a powerful window into ion transport mechanisms. Looking forward, their application can generate “molecular movies” of ions traversing soft and 2D membranes, illuminating how nanoscale morphology governs ion selectivity and paving the way for better membrane designs.

### 5.2 Application of AI and ML to Understand Membranes

The integration of artificial intelligence (AI) and machine learning (ML) into membrane research presents transformative opportunities for advancing our understanding and development of membrane technologies. High‐throughput characterization techniques, which generate vast datasets on molecule and ion uptake, solute transport, and membrane interactions under various conditions, are ripe for AI and ML applications. These technologies can efficiently analyze complex datasets, uncovering patterns and correlations that might be missed by traditional methods. For instance, AI algorithms can predict membrane performance across different solute mixtures, concentrations, and pH levels, as well as under diverse driving forces such as electric fields, pressure, and concentration gradients. This predictive capability can significantly accelerate the design and optimization of membranes tailored for specific applications.

Moreover, AI and ML can facilitate the development of novel membrane materials by identifying optimal combinations of physicochemical properties that enhance selectivity, permeability, and durability. ML models can simulate membrane behavior under realistic conditions, providing insights into the molecular‐scale interactions that govern transport dynamics. These models can also be used to optimize fabrication processes, ensuring that membranes achieve desired structural and functional characteristics. Looking forward, AI and ML could enable the creation of adaptive membrane systems that respond dynamically to environmental changes or operational demands. Such systems could autonomously adjust their properties to maintain optimal performance, reducing the need for manual intervention and extending membrane lifetimes. Additionally, AI‐driven platforms could integrate real‐time data from membrane operations to continuously refine and improve membrane designs, leading to more efficient and sustainable separation processes.

## Conflict of Interest

The authors declare no conflict of interest.
